# Upgraded Three-Wavelength Lidar for Real-Time Observations of Volcanic Aerosol Optical and Microphysical Properties at Etna (Italy): Calibration Procedures and Measurement Tests

**DOI:** 10.3390/s24061762

**Published:** 2024-03-08

**Authors:** Matteo Manzo, Gianpiero Aiesi, Antonella Boselli, Salvatore Consoli, Riccardo Damiano, Guido Di Donfrancesco, Benedetto Saraceno, Simona Scollo

**Affiliations:** 1 Dipartimento di Fisica “Ettore Pancini”, Università di Napoli Federico II, Complesso Universitario di Monte S. Angelo, 80126 Napoli, Italy; matteo.manzo@unina.it (M.M.); riccardo.damiano@unina.it (R.D.); 2 Istituto Nazionale di Geofisica e Vulcanologia, Osservatorio Etneo, Piazza Roma 2, 95125 Catania, Italy; gianpiero.aiesi@ingv.it (G.A.); salvatore.consoli@ingv.it (S.C.); benedetto.saraceno@ingv.it (B.S.); simona.scollo@ingv.it (S.S.); 3 Consiglio Nazionale delle Ricerche, Istituto di Metodologie per l’Analisi Ambientale (IMAA-CNR), 85050 Tito Scalo, Potenza, Italy; 4 Agenzia Nazionale per le Nuove Tecnologie, l’Energia e lo Sviluppo Economico Sostenibile (ENEA)—Unità Tecnica Antartide C.R. Casaccia, 00123 Roma, Italy; guido.didonfrancesco@enea.it

**Keywords:** lidar, aerosol parameters, calibration

## Abstract

An innovative mobile lidar device, developed to monitor volcanic plumes during explosive eruptions at Mt. Etna (Italy) and to analyse the optical properties of volcanic particles, was upgraded in October 2023 with the aim of improving volcanic plume retrievals. The new configuration of the lidar allows it to obtain new data on both the optical and the microphysical properties of the atmospheric aerosol. In fact, after the upgrade, the lidar is able to measure three backscattering coefficients, two extinction coefficients and two depolarisation ratios in a configuration defined as “state-of-the-art lidar”, where properties such as particle size distribution and the refractive index can be derived. During the lidar implementation, we were able to test the system’s performance through specific calibration measurements. A comparison in an aerosol-free region (7.2–12 km) between lidar signals at 1064 nm, 532 nm and 355 nm and the corresponding pure molecular profiles showed a relative difference of <1% between them for all the wavelengths, highlighting the good dynamic of the signals. The overlap correction allowed us to reduce the underestimation of the backscattering coefficient from 50% to 10% below 450 m and 750 m at both 355 and 532 nm, respectively. The correct alignment between the laser beam and the receiver optical chain was tested using the signal received from the different quadrants of the telescope, and the relative differences between the four directions were comparable to zero, within the margin of error. Finally, the first measurement results are shown and compared with results obtained by other instruments, with the aim of proving the ability of the upgraded system to more precisely characterise aerosol optical and microphysical properties.

## 1. Introduction

During explosive eruptions, volcanic plumes produce particles of different sizes and compositions named tephra, which are formed from the fragmentation of magma [1]. These particles have different sizes, ranging from a few microns (volcanic aerosol < 32 μm) to bombs/blocks > 64 mm. Volcanic plumes also contain water vapour and sulphur dioxide (SO_2_), which modify radiative forcing. In fact, sulphur dioxide (SO_2_) aerosol may scatter solar radiation, reducing the heating of the Earth’s surface and of the atmosphere below volcanic plumes [2]. The injection of volcanic ash or gas into the stratosphere has implications on climate change [3]. How volcanic plume affects the atmosphere is a function of the eruptive intensity and, hence, of the maximum height reached by the eruption column. In general, Plinian eruptions generate strong plumes that reach the stratosphere [4], whereas eruptions from Hawaiian to Strombolian form weak plumes located in the troposphere [5]. Whereas ground-based lidars are not able to detect strong plumes, weak plumes have a height under 10 km and are suitable for lidar measurements.

Volcanic particles formed during an eruption can constitute one of the most important volcanic hazards. In fact, they are dangerous for aviation operations, causing—in the worst case—aircraft engine failure [6]. Moreover, tephra fallout can affect some infrastructures [7] and cause diseases [8]. Fine volcanic ash (tephra with a size less than 63 μm), blown by winds, can reach long distances (in the order of several hundred kilometres [9]), also affecting areas far away from the eruptive vents [10] and involving different countries. Mainly for these reasons, the quantification of volcanic ash concentrations and tephra loads in the atmosphere is very important. These are usually provided by volcanic ash advisory centres (VAACs) or by volcano observatories [11] using volcanic ash transport and deposition models (VATDMs). However, VATDMs require the estimation of eruption source parameters and, consequently, volcanological observations are needed.

Measurements of eruption source parameters using different systems surrounding active volcanoes are crucial. The main eruption source parameters are the column height, mass eruption rate (or total mass) and total grain size distribution. Column height is the easiest to detect by means of remote sensing systems, spanning from visual observations and satellite images to radars [12] and lidars [13]. On the other hand, although the mass eruption rate has a first-order effect on dispersal and sedimentation, it is difficult to detect in real time and large uncertainties remain even when using inversion models [14]. Furthermore, measurements of the whole size spectrum of particles ejected during an eruption are possible only using remote sensing systems at different wavelengths. As these measurements are very difficult, grain size distribution as a whole is often assumed to be equal to some past eruptions or is based on an average of the measurements made in volcanic plumes [15].

During recent years, lidar measurements have become a valuable tool for monitoring volcanic plumes. As an example, lidar measurements from different sites were available during the 2010 Eyjafjallajökull eruption [15,16,17,18] and during the 2002 Etna eruptions [19]. The lidar network allowed for the estimation of important features of volcanic plume dispersal, such as its 4D distribution in the troposphere over Europe and its optical properties [20]. At Etna, lidar measurements have been carried out since 2010, initially using a single-wavelength lidar prototype [21] and then using a more complex multi-wavelength system [22]. These measurements allowed, for the first time, the measurement of the lidar ratio during an event and enabled more reliable estimates of volcanic ash concentration. The system has been recently updated to provide more detailed information on volcanic plumes at Etna. In this paper, we describe the improvements made to this system, its new calibration measures and future capabilities.

## 2. Materials and Methods

The VULCAMED project was developed under the National Operational Programme “Research and Competitiveness” 2007–2013 and was aimed at increasing the structural strengthening of research centres such as the Istituto Nazionale di Geofisica e Vulcanologia (INGV) in order to improve studies of high-risk volcanic areas and their geothermal potential in the Mediterranean. Within the VULCAMED project, an innovative lidar system was developed. The lidar was first designed to make measurements in the UV (355 nm, 386 nm) and IR (1530 nm) spectral regions but, successively, in 2023, it was upgraded to include elastic channels at 1064 nm and 532 nm, the N_2_ Raman channel at 607 nm and the H_2_O Raman channel at 407 nm, thanks to INGV’s funds received from PON-GRINT. Moreover, the parallel and perpendicularly polarised components (P and S) of the elastic signals at 355 and 532 nm were added in order to obtain a more accurate estimation of the detected aerosol, retrieving information about its shape. These new system features allow a complete aerosol characterization during volcanic activity.

The lidar system uses a compact diode pumped and air-cooled Nd:YAG laser (WEDGE model specifically developed by Bright Solutions s.r.l., Pavia, Italy). The laser emits 3 different wavelengths simultaneously: the fundamental (1064 nm), the second (532 nm) and the third harmonic (355 nm), with a 1 KHz repetition rate and output optical powers of 1 W, 1.5 W and 0.6 W, respectively.

**Figure 1:**
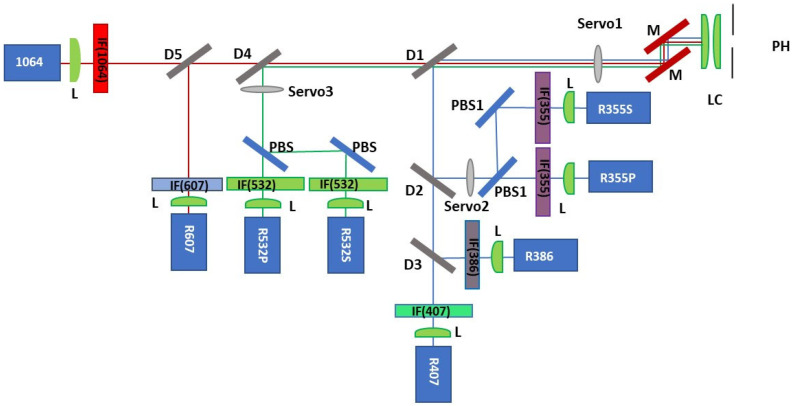
Experimental set-up of the polychromator unit after the lidar system upgrade: P stands for ‘Pinhole’, M for ‘Mirror’, D for ‘Dichroic mirror’, PBS for ‘Polarizing Beam Splitter’, L for ‘Lens’, IF (λ nm) for ‘Interferential Filter’. The servos (1–3) are used to insert the attenuators or the depolarizer plates along the optical path.

A Cassegrain telescope in a Dall–Kirkham configuration with a 35 cm diameter and F-number of 4.5 collects the backscattered radiation, which is then spectrally separated in an eight-channel polychromator unit by means of beam splitters and dichroic mirrors. Moreover, the system allows polarization measurements at both 355 and 532 nm. To accomplish this measurement, polarizing beam splitters are located inside the polychromator unit in order to split the light into its parallel and perpendicularly polarized components (P and S). Narrow bandpass filters produced by ALLUXA Inc. (USA) are located in front of the detectors, allowing for the selection of specific wavelengths. The signals are detected, for each channel, using photomultiplier tubes (Hamamatsu H10721P-210 for 355, 386, 407, 532 nm and H16721 for 607 nm), except for the 1064 nm channel, where an avalanche photodiode (APD-3.0 LICEL GmbH, Berlin, Germany) in an analog regime is used. The experimental set-up of the lidar polychromator unit is illustrated in Figure 1. The system parameters are summarized in Table 1. 

Detected signals are processed by a sophisticated and high performing data acquisition system (ALA CLASS Configurable Lidar Acquisition SyStem) designed by ALA Advanced Lidar Applications s.r.l.. It includes a motherboard and independent acquisition modules for photon counting (up to 5 analog inputs, 16 Kbins, 10–1000 ns temporal resolution, photo-rate up to 200 MHz, trigger rate up to 10 KHz). A single electronic board (ALA LARA LidAR controller boArd) allows for the management of the Lidar system by means of an intuitive and user-friendly software.

Optical properties of the aerosol, such as the backscattering coefficient (β), the extinction coefficient (α) and the aerosol depolarization ratio (δ), are retrieved from the lidar data using inversion algorithms. In particular, β was obtained using two different methods: the Klett–Fernald [23,24] for diurnal measurements, when only elastic signals are available, and the Elastic/Raman method [25] for measurements carried out after sunset. The first method requires an assumption on the ratio between α and β, the Lidar Ratio LR. The α coefficient was retrieved using the inversion method proposed by Ansmann [26], while δ was calculated using the ratio between the P and S backscattering coefficients [27]. Finally, the water vapor mixing ratio was retrieved from the 407 nm Raman signal, using the correspondent N_2_ Raman signal as a reference [28,29].

The lidar, in this new configuration, is able to measure three β, two α and two δ, allowing the retrieval of the aerosol’s optical and microphysical properties. The system upgrade brought the lidar to a configuration defined as “state-of-the-art lidar”, according to [30], whose properties such as volume particle size distribution and refractive index of the particles can be derived.

## 3. Results

### 3.1. Calibration Methods

The optimization of the results achieved from a lidar instrument depends on the implementation of specific measurement tests to establish the system’s performance and to define a number of technical parameters, allowing for a reduction in uncertainty for the retrieved profiles. In order to make accurate measurements of the aerosol’s optical and microphysical properties, the lidar system should then be calibrated. The system calibration concerned different issues: the checking of the lidar signal dynamic range; the measurement of the Gain ratio (G) for the depolarization calibration and of the overlap function (O(z)) to correct the lidar signal at lower altitudes; the analysis of the near-range signal to test the optical and optomechanical design of the lidar receiver; the multiwavelength channel calibration.

#### 3.1.1. Rayleigh Fit Test

The comparison between the lidar profile and the one expected from a pure molecular atmosphere, the latter derived from the air density and temperature profiles obtained by nearby radio-sounding or by standard atmosphere look-up table, allows for the verification of the correct dynamics of the lidar signal. The procedure, known as a Rayleigh fit test, is based on the normalization between the two profiles in clear atmospheric conditions and is used in lidar data pre-processing in order to check the correct background noise that is to be subtracted from the signal before data retrieval.

**Figure 2:**
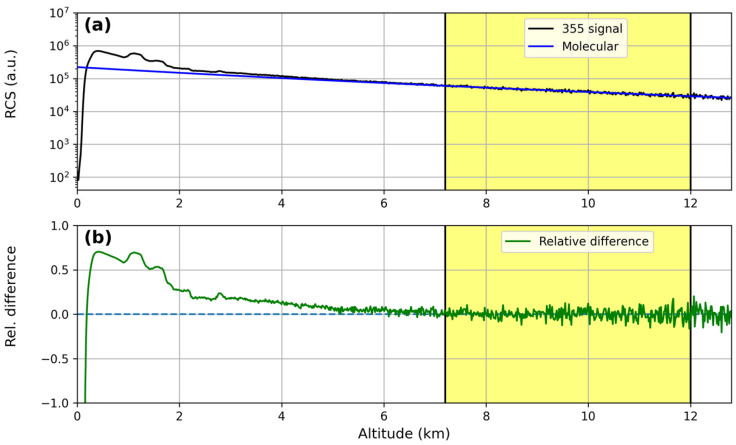
Results of Rayleigh fit tests for the 355 nm wavelength: (**a**) fit between the Range-Corrected lidar Signal (RCS) and a pure molecular profile; (**b**) relative differences between the RCS and the pure molecular profile.

**Figure 3:**
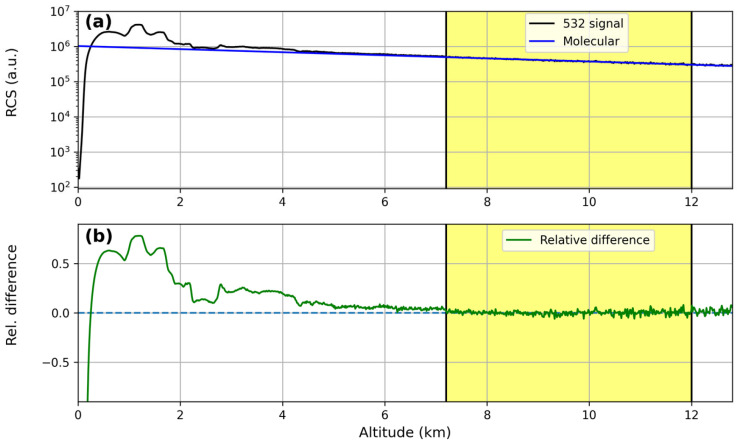
Results of Rayleigh fit tests for the 532 nm wavelength: (**a**) fit between the Range-Corrected lidar Signal (RCS) and a pure molecular profile; (**b**) relative differences between the RCS and the pure molecular profile.

**Figure 4:**
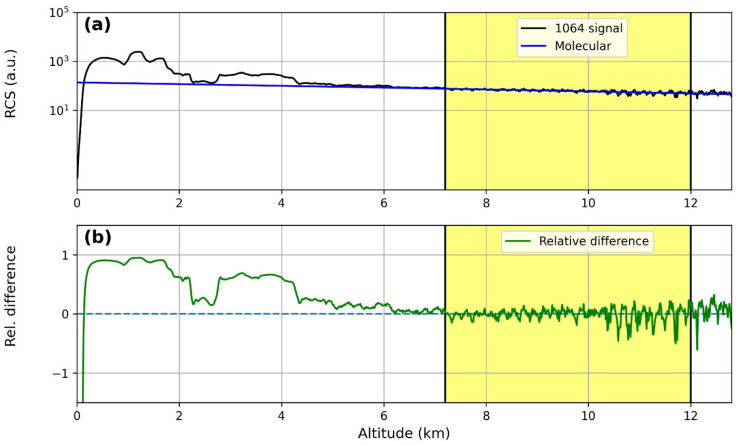
Results of Rayleigh fit tests for the 1064 nm wavelength: (**a**) fit between the Range-Corrected lidar Signal (RCS) and a pure molecular profile; (**b**) relative differences between the RCS and the pure molecular profile.

A comparison between the Range-Corrected Signal (RCS-black line) of the lidar and the molecular profile (blue line) at the three different wavelengths (355, 532 and 1064 nm) is reported in Figure 2a, Figure 3a and Figure 4a; Figure 2b, Figure 3b and Figure 4b report the relative difference between them. Lidar signals were normalized to the molecular profiles in the region between 7.2 km and 12 km (yellow region in the figures), and for all the wavelengths, the relative differences between them were less than 1%, highlighting the correct lidar signal dynamics.

#### 3.1.2. Depolarization Calibration

The lidar is able to detect the components of the signals at 355 nm and 532 nm parallel (P) and perpendicular (S) with respect to the polarization plane of the emitted laser beam. A depolarization calibration procedure is needed to estimate the gain factor between these two lidar channels for each wavelength.

The parameter G, called the gain ratio, depends on the gain difference between the two channels and must be measured to retrieve the corrected aerosol linear depolarization ratio δ [27,31,32].

In order to make the backscattered light fully unpolarized, a depolarizer plate was placed inside the polychromator unit along the optical path of each of the two branches, corresponding to 355 nm and 532 nm. In addition, a correction using the pure molecular scattering profile is required, due to the not-perfectly polarized light coming out from the laser and the various optical components on the beam optical path that can introduce a depolarization factor.

This calibration method followed the two-step procedure detailed in [33]: firstly, the gain ratio between the two channels was evaluated, and then, the correction of the instrument depolarization was achieved using pure molecular scattering profiles, normalizing the gain ratio to an appropriate value known from theory [31].

**Figure 5:**
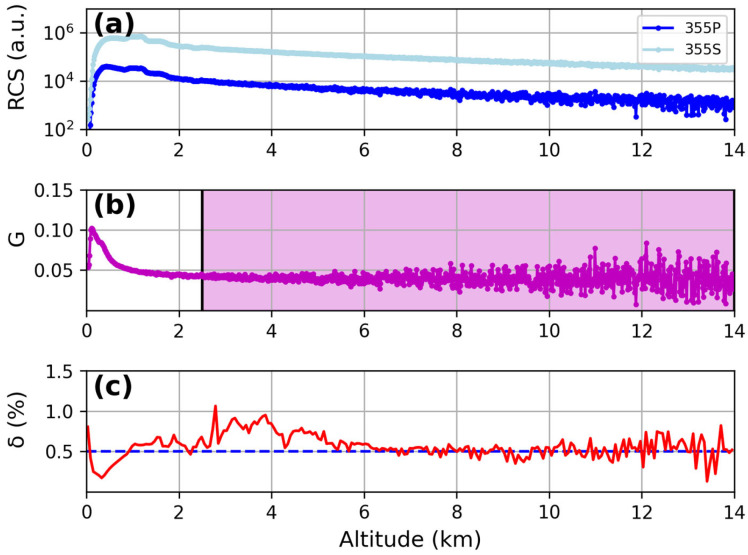
Results for the depolarization calibration at 355 nm: (**a**) comparison between P (blue line) and S (light blue line) signals in a series of measurements with the depolarizer plate; (**b**) gain ratio at 355 nm (violet line); (**c**) total depolarization ratio percentage at 355 nm (red line). The average value of δ is about 0.5% in the aerosol-free region 8–14 km.

As shown in Figure 5, the mean value of δ at 355 nm between 8 km and 14 km, corresponding to an aerosol-free region, was equal to (0.51 ± 0.11)%, in agreement with the expected depolarization of 0.5% for a pure molecular scattering signal, according to the bandwidth of the used interference filter [31].

**Figure 6:**
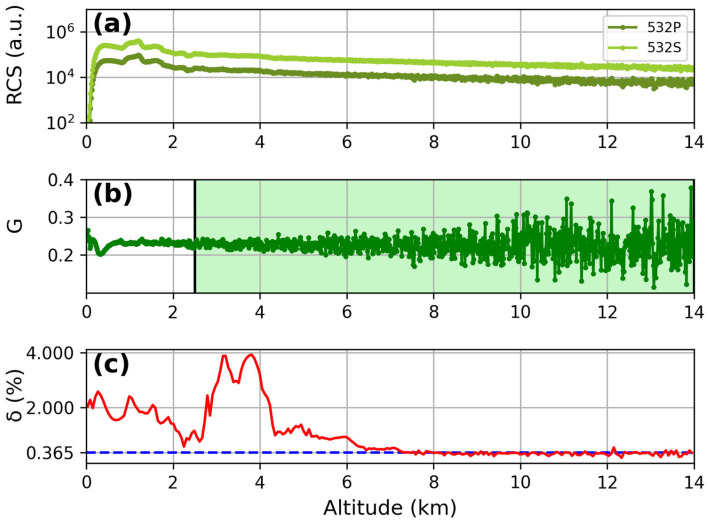
Results for the depolarization calibration at 532 nm: (**a**) comparison between P (green line) and S (light green line) signals in a series of measurements using the depolarizer plate; (**b**) gain ratio at 532 nm (dark green line); (**c**) total depolarization ratio percentage at 532 nm (red line). The average value of δ was about 0.37% in the aerosol-free region of 8–14 km.

The same procedure was performed for the 532 channels (Figure 6). In this case, the mean δ value, in the aerosol-free range of 8–14 km, resulted in (0.32 ± 0.05)%; this is comparable, within the margin of errors, to the value of 0.365% expected in theory for pure molecular scattering signal at 532 nm, according to the bandwidth of the used interference filters [31].

#### 3.1.3. Overlap Function

**Figure 7:**
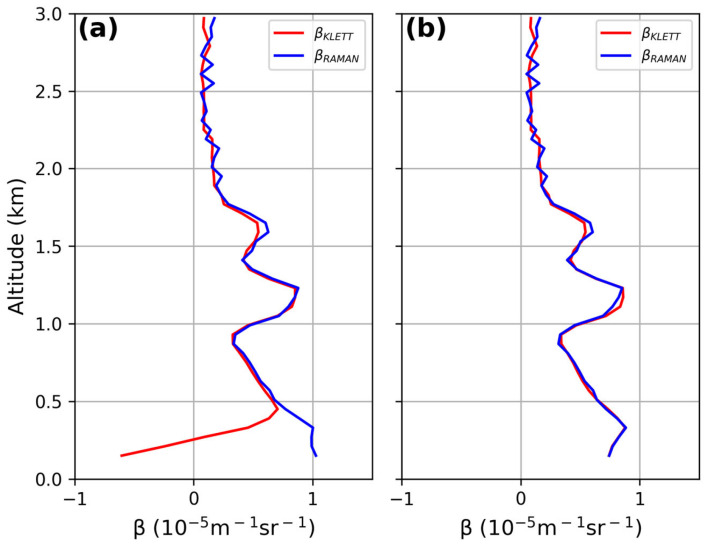
Comparison between β_KLETT_ and β_RAMAN_ at 355 nm before and after overlap correction: (**a**) backscattering coefficients before overlap correction; (**b**) backscattering coefficients matching after just 2 iterations of the method.

**Figure 8:**
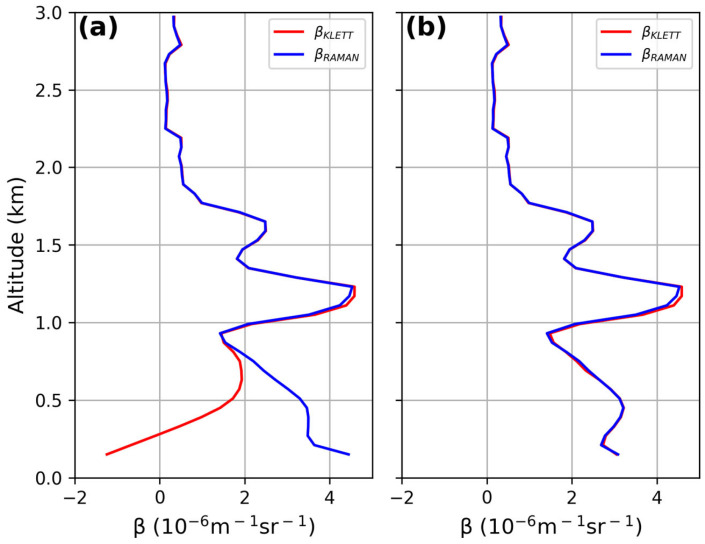
Comparison between β_KLETT_ and β_RAMAN_ at 532 nm before and after overlap correction: (**a**) backscattering coefficients before overlap correction; (**b**) backscattering coefficients matching after 2 iterations of the method.

The lidar signal coming from altitudes close to the ground for a bistatic system is often underestimated due to the incomplete overlap between the laser beam and the telescope’s field of view (FOV). Correcting the signal with the overlap function down to very low altitudes allows useful data to be obtained for lower-atmosphere investigation and air quality control. A good overlap correction also affects the lidar-derived particle size distribution, because aerosol is mainly contained within the lowest troposphere. To determine the overlap function, among other possible methods [33,34,35,36], there is an iterative method using both Raman and Elastic backscattered signals that was applied here. Here, we make the assumption that the lidar ratio is constant within the first kilometers of the atmosphere or, in other words, that the aerosol typology does not change. The used method is detailed in [33]. Figure 7a and Figure 8a show the overlap function affecting the aerosol backscattering profiles derived by the Klett inversion method (red line in the figures); conversely, the aerosol backscattering coefficient retrieved from the ratio between the elastic and the Raman lidar profiles (blue line in the figure) is not dependent on the overlap function. The iterative procedure allowed for the correction of the aerosol profile for the overlap function after just two iterations and, as expected, both the derived backscattering profiles were superimposed (Figure 7b and Figure 8b). The overlap correction allowed for a reduction of the underestimation of the backscattering coefficient from 50% to 10% below 450 m and 750 m at both 355 and 532 nm, respectively.

**Figure 9:**
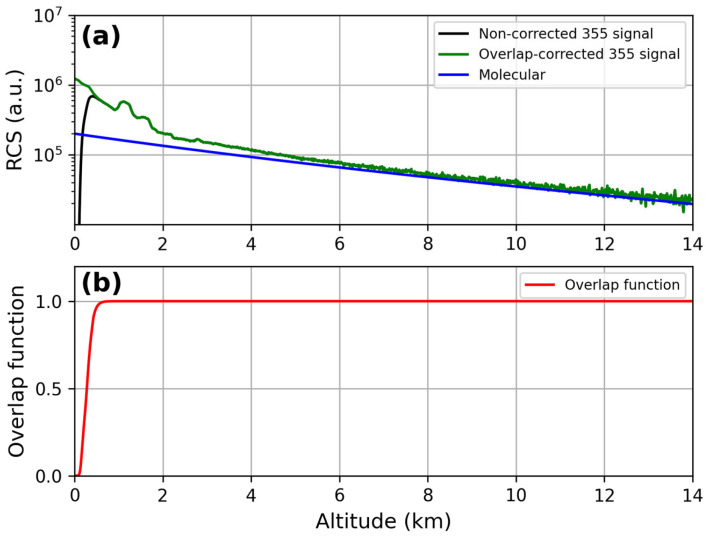
(**a**) Comparison between RCS at 355 nm before and after the overlap correction; (**b**) overlap function at 355 nm.

**Figure 10:**
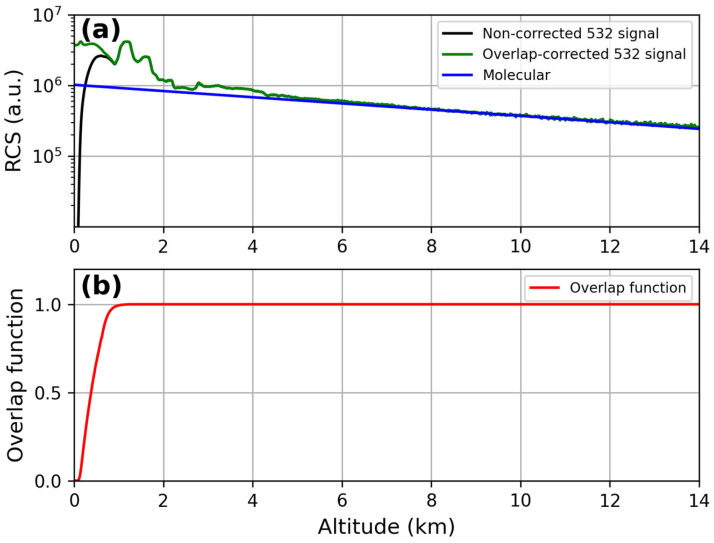
(**a**) Comparison between RCS at 532 nm before and after the overlap correction; (**b**) overlap function at 532 nm.

The lidar signals, before and after the overlap correction, are reported in Figure 9a and Figure 10a at 355 nm and 532 nm, respectively. The corresponding overlap functions resulting from the iterative procedure are displayed in panel (b) of each figure: full overlap heights of 580 m for the lidar signal at 355 nm and of 900 m for the lidar signal at 532 nm were obtained.

#### 3.1.4. Telecover Test

The Telecover test allows for the verification of the correct alignment between the laser beam and the receiver optical chain by checking the signal received from the different quadrants of the telescope.

The Rayleigh fit works in the far-range, where it is likely to find a portion of the lidar profile in clear air. On the other hand, there is no calibration method for the near-range, where the lidar profile is likely characterized by a stable presence of urban and natural pollution. Unfortunately, it is at low altitudes that the effects of misalignments and shortcomings of the optics show the most [37]. To overcome this problem, it is necessary to perform a test in which the lidar signals, acquired using different sectors of the telescope, are compared. If the optical alignment of the lidar system is correct, it is expected that the signals from different sectors do not show any difference after normalization. The comparison, hence, suggests a complete overlap.

The method used in this work is the Quadrant test [37], where the telescope is covered in such a way that only a quarter of it is used at a time. Measurements of 15 min for each quarter of the telescope were carried out, repeating the measurement of the first quadrant at the end, with the aim of taking into account any changes in the atmospheric profile during the test.

**Figure 11:**
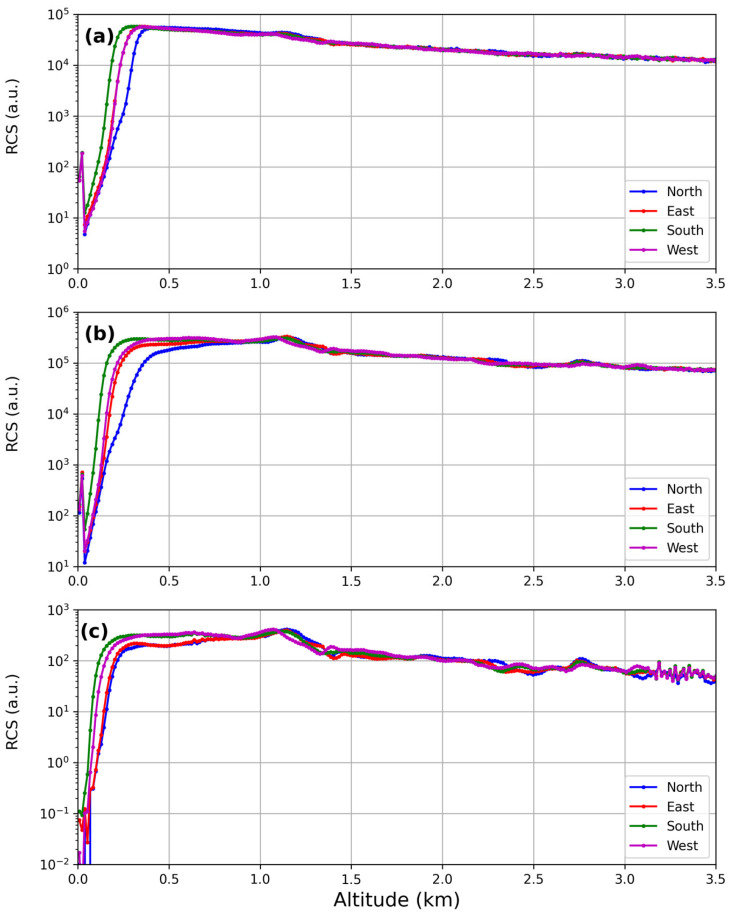
The Telecover test at 355 nm (**a**), 532 nm (**b**) and 1064 nm (**c**). The different colours identify different sectors of the telescope.

The results of the Telecover test for the lidar are reported in Figure 11.

Normalized Lidar signals did not show differences at higher altitudes; the relative difference between the RCS signals corresponding to each channel for the four directions (W–N–E–S) resulted around zero in the range 1–3.5 km, as shown in Table 2.

The results of the Telecover test suggest that the full overlap distance at 355 and 532 nm is the one estimated in Section 3.1.3. The same figure depicts that the 1064 nm signal shows full overlap at about 900 m, similar to the 532 nm channel.

#### 3.1.5. Channel Calibration

With the aim of verifying the correct wavelength dependence of the measured optical parameters, we calculated the Backscatter-related Angstrom Exponents (BAE) measured at the different wavelengths.

This parameter is expressed as:

$$BAE=-\frac{\log\left(\frac{\beta \left(\lambda_{i}\right)}{\beta \left(\lambda_{j}\right)}\right)}{\log\left(\frac{\lambda_{i}}{\lambda_{j}}\right)}$$

where λ_i/j_ are the emitted wavelengths (355, 532 and 1064 nm) and β is the corresponding aerosol backscattering parameters.

**Figure 12:**
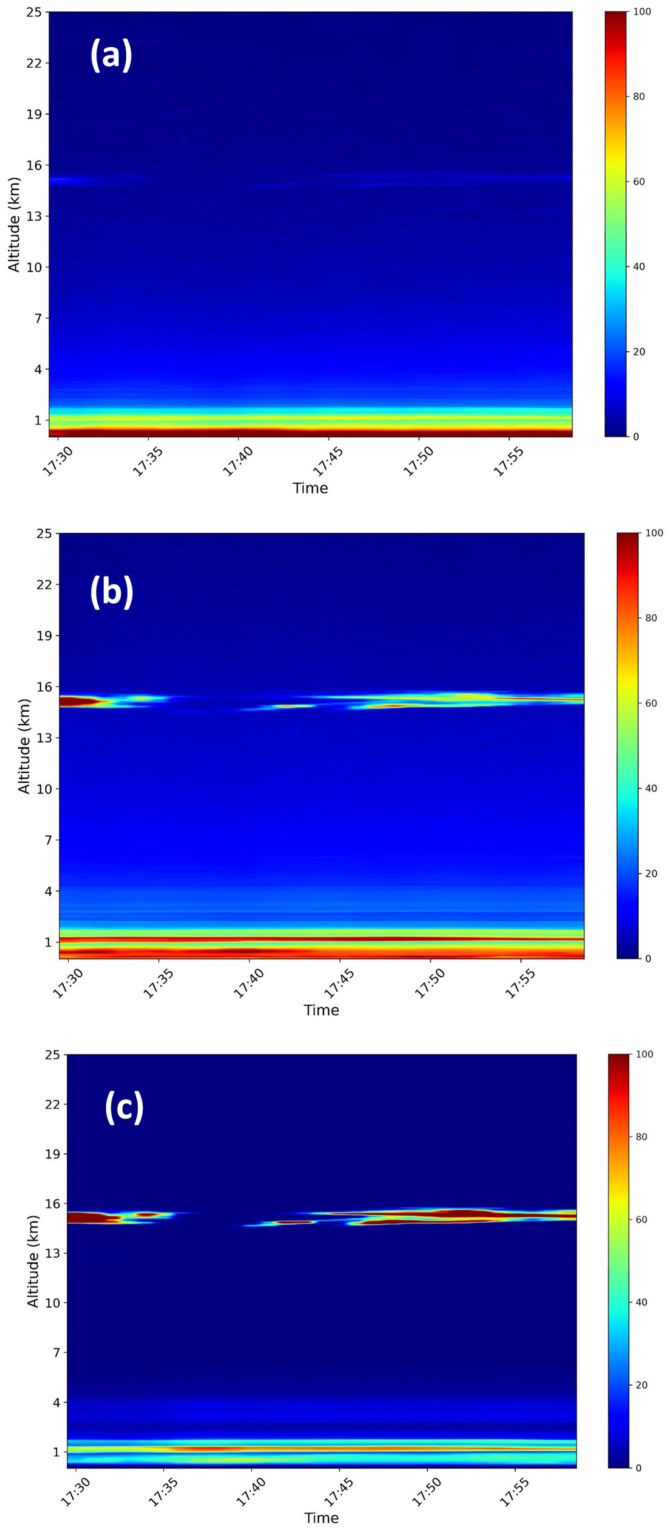
Colour maps of the lidar signals measured at 355 nm (**a**), 532 nm (**b**) and 1064 nm (**c**), showing the spatio-temporal variation of a thin cirrus layer observed in the atmospheric column up to 25 km of altitude. The colour scale units are arbitrary (a.u.).

A lidar profile characterized by a thin cirrus cloud is a good test to verify that the calculated BAEs at each of the three wavelengths show the same value inside the cloud layer, the backscattering coefficient being almost independent of the wavelength, in agreement with values reported in other works for the same wavelength couples [38,39,40,41]. For this aim, Lidar measurements carried out on 5 October 2023 from 17:30 to 18:00 UTC, showing a cirrus at about 15 km of altitude, were used. The vertical and temporal resolutions of the measure were of 15 m and 60 s, respectively. Figure 12 reports the colour maps of the range-corrected lidar signals (RCS), showing the spatio-temporal variation of the aerosol layering observed in the atmosphere at the three wavelengths.

**Figure 13:**
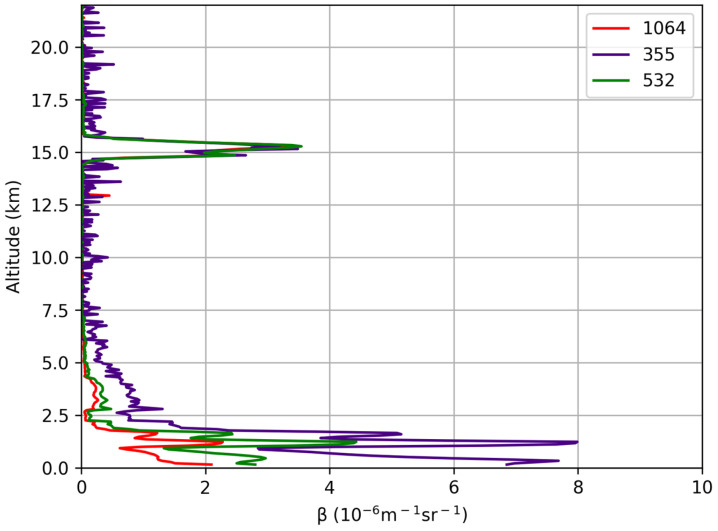
Aerosol backscattering coefficient profiles measure at 355 nm (purple line), 532 nm (green line) and 1064 nm (red line) from 17:30 to 18:00 UTC.

The backscattering coefficients at each wavelength, retrieved with a spatial resolution of 30 m, are reported in Figure 13. The profiles at different wavelengths appear almost superimposed in the atmospheric range between 14 and 16 km, where the cirrus cloud is detected. As expected, the three BAE values summarized in Table 3 were comparable with zero, hence showing a correct calibration between the channels.

#### 3.1.6. Water Vapour Mixing Ratio Measurement

**Figure 14:**
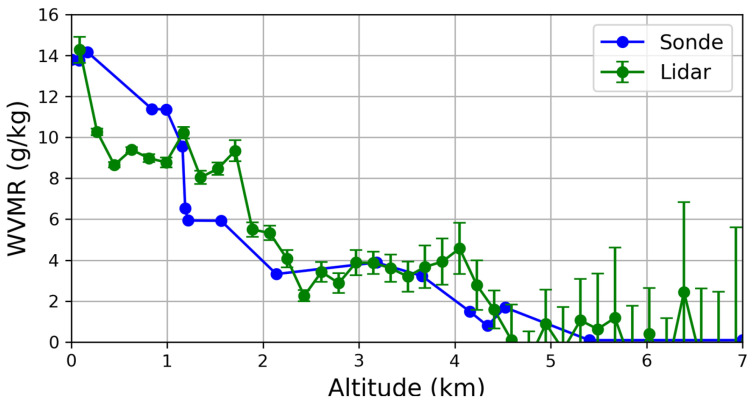
Water Vapour Mixing Ratio (g/Kg) derived by Raman lidar measurements (green line) and radiosounding data from Pratica di Mare (blue line). Error bars are reported only from lidar data due to the lack of the corresponding information for the radio-sounding data.

The lidar echoes at 386 nm and 407 nm were used to obtain the water vapor mixing ratio (WVMR) profile by means of the Raman technique [28]. This approach allows the WVMR to be obtained from the ratio between the two lidar signals, assuming a calibration constant that can be determined comparing the lidar results with the radiosonde-derived WVMR profiles. The closeness of the airport to the lidar location does not allow the balloon-borne radiosonde to be used. Therefore, we used Pratica di Mare radio-sounding data taken at the time closest to the lidar observations, i.e., at 00:00 UTC. Figure 14 shows the comparison between the calibrated WVMR profile derived from lidar and the one measured by the radiosonde. Even if the system allows for probing of the atmosphere up to 15 km during the day and up to 20 km at night, the water vapor signal—due to the lowest Raman cross-section of H_2_O molecules—reaches approximately 7–8 km in the evening. For this reason, a profile for up to 7 km for water vapor has been shown.

Despite the large distance between the two observational sites (about 240 km because the measurement tests were carried out in Naples) and the time difference between the measured profiles (about 6 h), there was good agreement, providing evidence of a correct spectral selection in the design of the lidar receiver.

#### 3.1.7. Lidar-Derived Size Distribution

The aerosol optical properties measured by the lidar at different wavelengths (three β and two α profiles) were used to retrieve the volume particle size distribution *dV*(*r*)/*dln*(*r*) (expressed in a.u.). This was achieved using our inversion procedure based on a Bayesian model run with Monte Carlo simulations [42]. Specifically, we used averaged values of β(z) and α(z) over the whole measured atmospheric column with the aim of comparing the lidar-derived size distribution with the columnar size distribution provided by the AERONET sun-photometer data. As the tests were carried out in the Naples ACTRIS National Facility, located at CeSMA (Centro Servizi Metrologici e tecnologici Avanzati), the involved sun-photometer was sited there (https://aeronet.gsfc.nasa.gov/cgi-bin/bamgomas_interactive, accessed on 17 January 2024).

**Figure 15:**
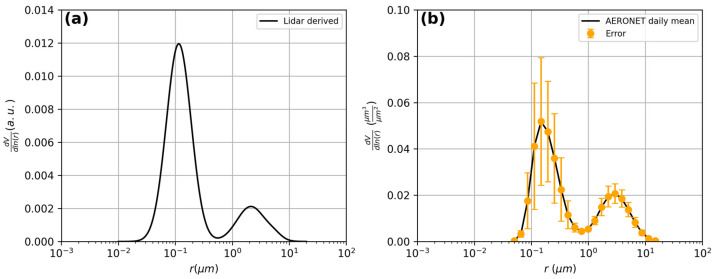
Particles size distributions retrieved by a co-located AERONET sun-photometer (**a**) and Lidar (**b**) measurements.

The lidar-derived size distribution is reported in Figure 15a. The size distribution was obtained from lidar data measured at 18:00 UTC and shows a bimodal shape with radius values of about 0.11 µm and 2.12 µm.

The lidar-derived size distribution showed fairly good agreement with the columnar particle size distribution provided by the AERONET sun-photometer, as reported in Figure 15b. In this Figure, the columnar size distribution, averaged over the day, is shown together with the mean standard deviation that was used for data uncertainty. In particular, for the AERONET data measured at 14:19 UTC, the size distribution was bimodal, with the two mode radius values at about 0.15 µm and 2.94 µm, respectively. In our opinion, the differences between the mode radii of the two distributions are likely due to the different measurement times.

## 4. Conclusions

A mobile Lidar system has been upgraded, tested and calibrated. The aim of such a new configuration, with the added capability of measuring three β, two α and two δ, was to increase the potential of the system to retrieve the optical and microphysical properties of the aerosol. The tests performed after specific calibration procedures showed a relative difference of less than 1% for all the wavelengths between the calibrated RCS and the molecular profile in the region 7.2–12 km (assumed aerosol-free atmosphere). In the same atmospheric region, the averaged molecular depolarization values at 532 nm and 355 nm were, after the channels’ calibration, equal to (0.32 ± 0.05)% and (0.51 ± 0.11)%, respectively. The N_2_ Raman channels allowed for the measurement of the full overlap height of the system—that is, 580 m at 355 nm and 900 m at 532 nm—while the results of the standard Quadrant-test method allowed for the estimation of a value of 900 m for the IR signal.

The retrieved WVMR and volume particle size distribution were compared with those obtained by different instrumentation. The relative differences between the lidar-retrieved WVMR and the radio-sounding data from Pratica di Mare, in the atmospheric region of 0.1–4 km, were less than 10%; these are in reasonable agreement, considering the large spatial and temporal distances between the two measures.

Using the spectral aerosol optical properties measured by lidar (three β and two α profiles) at 18:00 UTC, the volume particle size distribution was obtained and compared to the columnar size distribution measured at 14:19 UTC by the AERONET sun-photometer of the ACTRIS National Facility of Napoli CeSMA. Both lidar and AERONET measurements indicated a bimodal shape, with radius values of about 0.11–2.12 µm and 0.15–2.94 µm, respectively.

The reasonable agreements of both the retrieved WVMR and size distribution, along with the very good lidar performances in terms of signal linearity, polarization and spectral calibration, indicate the ability of the upgraded system to more precisely characterize the aerosol optical and microphysical properties, bringing the lidar to a configuration definable as “state-of-the-art lidar”. The new system configuration will allow for a comprehensive characterization of volcanic aerosol, enabling the profiling of its microphysical characteristics; this possibility has not yet been realized in the monitoring and study of volcanic eruptions.

## Figures and Tables

**Table 1:** Main parameters of the lidar system.

**Laser Source**	
Diode Pumped Nd:YAG	Fundamental, 2nd and 3rd harmonics
Repetition Rate	1 KHz
Peak Power	1 W at 1064 nm, 1.5 W at 532 nm, 0.6 W at 355 nm
Pulse Width	1 ns
Linear Polarization	>100:1
**Receiver System**	
Telescope Diameter	35 cm
Telescope Field of View	1 mrad
Elastic Channels	355 nm (P/S), 532 nm (P/S), 1064 nm
Raman Channels	386 nm, 407 nm, 607 nm
Interferential Filters FWHM	at 355 nm, 532 nm, 386 nm 0.5 ± 0.1 nm at 407 nm, 607 nm, 1064 nm 1 ± 0.1 nm

**Table 2:** Relative difference between the RCS signal received from the West (W) quadrant and the other directions (North (N), South (S) and East (E)) at different wavelengths. The reported errors were calculated as the standard deviation.

	355 nm	532 nm	1064 nm
**(W-N)/W**	−0.03 ± 0.08	−0.01 ± 0.10	−0.002 ± 0.20
**(W-E)/W**	−0.01 ± 0.05	−0.005 ± 0.100	0.03 ± 0.20
**(W-S)/W**	−0.004 ± 0.040	0.005 ± 0.060	0.02 ± 0.10

**Table 3:** Backscatter-related Angstrom exponent obtained at different wavelengths (355 nm, 532 nm and 1064 nm).

λ_i_/λ_j_	BAE
355/532	0.03 ± 0.13
355/1064	0.06 ± 0.05
532/1064	0.08 ± 0.06

## Data Availability

Dataset available on request from the authors.
